# Infrared and Visual Image Fusion Based on a Local-Extrema-Driven Image Filter

**DOI:** 10.3390/s24072271

**Published:** 2024-04-02

**Authors:** Wenhao Xiang, Jianjun Shen, Li Zhang, Yu Zhang

**Affiliations:** 1 Department of Electronic Engineering, Tsinghua University, Beijing 100084, China; xiangwh2018@163.com (W.X.); sjj20@mails.tsinghua.edu.cn (J.S.); chinazhangli@tsinghua.edu.cn (L.Z.); 2 School of Astronautics, Beihang University, Beijing 102206, China

**Keywords:** infrared and visual image fusion, local-extrema-driven image filter, bright feature map, dark feature map, base image

## Abstract

The objective of infrared and visual image fusion is to amalgamate the salient and complementary features of the infrared and visual images into a singular informative image. To accomplish this, we introduce a novel local-extrema-driven image filter designed to effectively smooth images by reconstructing pixel intensities based on their local extrema. This filter is iteratively applied to the input infrared and visual images, extracting multiple scales of bright and dark feature maps from the differences between continuously filtered images. Subsequently, the bright and dark feature maps of the infrared and visual images at each scale are fused using elementwise-maximum and elementwise-minimum strategies, respectively. The two base images, representing the final-scale smoothed images of the infrared and visual images, are fused using a novel structural similarity- and intensity-based strategy. Finally, our fusion image can be straightforwardly produced by combining the fused bright feature map, dark feature map, and base image together. Rigorous experimentation conducted on the widely used TNO dataset underscores the superiority of our method in fusing infrared and visual images. Our approach consistently performs on par or surpasses eleven state-of-the-art image-fusion methods, showcasing compelling results in both qualitative and quantitative assessments.

## 1. Introduction

The need for infrared and visible image fusion arises from the desire to obtain a comprehensive representation of a supervised scenario throughout the day. This technique finds extensive application in both civilian and military surveillance systems, as it can provide valuable information for decision making and situational awareness. Challenges in infrared and visible image fusion include precise segmentation of source images, the integration of salient features without the loss of visual information, and achieving a fusion image with high contrast and visual appeal. Traditional methods, such as spatial-domain and transform-domain approaches, often struggle with these challenges, resulting in suboptimal fusion effects. The motivation for infrared and visible image fusion lies in the complementary nature of the two imaging modalities. Infrared images capture thermal radiation emitted by objects, providing information about their temperature and potentially revealing hidden or camouflaged targets. Visible images, on the other hand, offer high-resolution detail and color information, facilitating the identification and recognition of objects and scenes. By fusing these two types of images, it is possible to achieve a more complete and accurate representation of the supervised scenario.

Various imaging sensors can capture different perspectives of a supervised scenario. The fusion of these multiple images proves invaluable in gaining a comprehensive understanding of the situation at hand [1,2,3]. For instance, the fusion of multi-modal medical images greatly aids surgeons in accurate disease diagnosis [4,5,6,7], while multi-focus image fusion yields a sharp, all-in-focus image [8,9,10,11,12]. In the realm of infrared and visual image fusion, it results in a composite image that provides a holistic representation of the supervised scenario throughout the day. This technique finds extensive application in both civilian and military surveillance systems [13,14,15,16,17]. Therefore, the development of innovative methods for fusing infrared and visual images is crucial and holds significant utility in both civil and military operations.

In recent years, the field of infrared and visual image fusion has witnessed the emergence of numerous methods, broadly categorized into spatial-domain and transform-domain approaches. Spatial-domain methods involve the initial segmentation of source images into multiple regions, followed by the combination of salient regions to achieve fusion [8,9,11,12,18]. However, these methods often struggle with precise segmentation, leading to suboptimal fusion effects. Transform-domain methods, gaining popularity over the past two decades, mainly include pyramid-based [19,20], wavelet-based [21,22], and sparse-representation-based image-fusion methods [23,24,25]. These methods extract salient features within a specific domain and integrate them to produce the fusion image, typically visually appealing, but susceptible to blurring or significant information loss.

In recent times, numerous deep learning approaches, particularly those based on convolutional neural networks (CNNs), have been proposed for image fusion [3,6,16,26,27,28,29,30]. Initially, Liu et al. [26] introduced a CNN model to identify the focus decision map of multi-focus images. They refined the focus decision map through post-processing procedures and generated an all-in-focus fusion image by copying focused regions from corresponding partially focused images based on the focus decision map. Subsequently, Li et al. [27] utilized densely connected CNN blocks to construct their image fusion model, achieving significant improvement in fusing infrared and visual images. Afterward, Ma et al. [16] employed a GAN-based model to effectively train their image fusion model for infrared and visual images in an adversarial manner. More recently, Li et al. [29] proposed a representation-learning-based infrared and visual image fusion network, claiming to avoid trial-and-test strategies. Despite their success in image fusion, most of these methods still exhibit low contrast or other types of defects.

In addition to the aforementioned methods, Zhou et al. [18] employed Gaussian and bilateral filters to extract multi-scale feature maps from different input images, subsequently blending them to create their fusion images. Similarly, Zhang et al. [31] devised a multi-scale Bezier filter, utilizing it to extract multiscale bright and dark features from infrared and visual images and integrating these features with the base image to generate their fusion image. Despite these efforts, their proposed image filters did not demonstrate sufficient superiority. Their image-fusion methods primarily concentrated on merging salient features without adequate consideration for the visual effect of the resulting fusion images. Consequently, their fusion images often suffered from low-contrast effects or the loss of visual information, making them unsatisfactory for human visual perception.

To address the limitations of existing methods and integrate the salient features of infrared and visual images while improving the visual quality of the fusion image, in this study, we introduce a simple, yet effective local-extrema-driven image filter. By alternately leveraging local minima and local maxima for image reconstruction, our proposed filter demonstrates exceptional capabilities in extracting both bright and dark features from images. Specifically, the disparities between the filtered and original images reveal these bright and dark features. Additionally, we present a multi-scale local-extrema-filter-based method for fusing infrared and visual images. This method initially extracts multiple scales of bright and dark feature maps and generates corresponding base images from the input infrared and visual images, respectively. It then merges the high-frequency bright and dark feature maps and low-frequency base images using two different fusion rules. Finally, the fusion image is generated by integrating the fused feature maps and the base image. Owing to the exploitation of our advanced local-extrema-driven filter, this method excels in capturing salient dark and bright features from both infrared and visual images, resulting in an informative fusion image. Moreover, the incorporation of our innovative structural similarity- and intensity-based base image fusion scheme enhances the visual quality of our fusion images, representing a notable improvement over current state-of-the-art image-fusion methods, including deep learning-based approaches.

This paper comprises three primary contributions. Firstly, we introduce an innovative image filter driven by local extrema, which effectively smooths images by removing bright and dark features, thus enabling robust feature extraction for generating salient bright and dark feature maps. Secondly, we propose a novel base image fusion scheme based on structural similarity and intensity considerations. This approach prioritizes obtaining a fused base image that encompasses large-scale structural features and well-distributed intensity, achieved through the generation of a weight map that accounts for these factors within the base images. Consequently, our method consistently produces fusion images with superior visual quality. Lastly, extensive experimental validation demonstrates the effectiveness of our approach, surpassing eleven state-of-the-art transform-domain image-fusion methods and outperforming leading deep learning-based methods. This success underscores the efficacy of our proposed local-extrema image filter and base image-fusion scheme.

The remaining paper is organized as follows. The proposed local-extrema-driven image filter and the proposed image-fusion method based on this filter are elaborated in Section 2. The experimental results and discussions are presented in Section 3. Finally, the conclusions of this paper are drawn in Section 4.

## 2. Proposed Method

**Figure 1:**
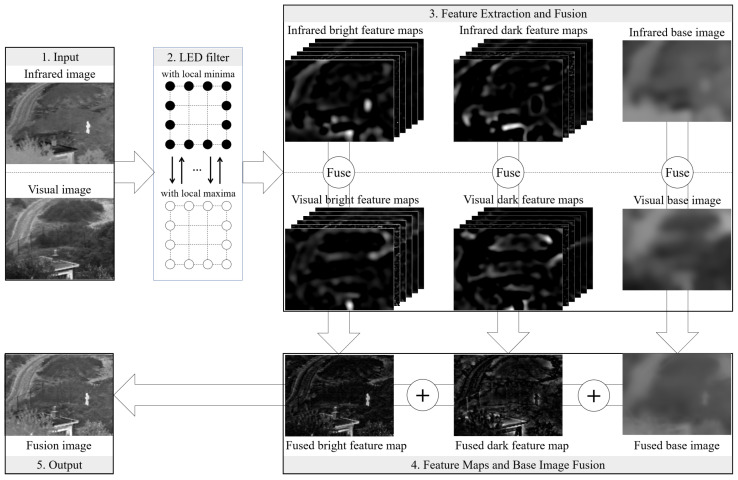
Flowchart of our proposed infrared and visual image-fusion method. Please note that, in order to visualize the dark feature maps (features with negative values), the absolute dark feature maps are presented in this figure. Moreover, the term “LED filter” is short for our proposed local-extrema-driven image filter.

In this study, we present an effective method for fusing infrared and visual images, leveraging our newly developed multi-scale local-extrema-driven image filter. The proposed approach comprises four key steps: Firstly, we apply the local-extrema-driven image filter at varying scales to progressively process the infrared and visual images. Simultaneously, we extract the corresponding bright and dark feature maps from each, while using the resulting filtered images as their base images. Next, we merge the bright and dark feature maps from both the infrared and visual images by selecting their elementwise maximum values, followed by enhancement with a scale-dependent coefficient. Then, we blend the base images of the infrared and visual inputs by a structural similarity-based fusion scheme. Ultimately, the fusion image is generated by integrating the fused bright and dark feature maps with the base image. To facilitate comprehension, we provide a flowchart of our proposed image method in Figure 1. In the following two subsections, the proposed image filter and image-fusion method based on this filter are elaborated, respectively.

### 2.1. Local-Extrema-Driven Image Filter

Within an image, bright features, as exemplified by the bright person in the infrared image shown in Figure 1, and dark features, represented by the dark window in the same infrared image, are commonly present. Employing a strategy of smoothing the image and subsequently subtracting the smoothed version from the original has proven to be an effective method for isolating the image’s bright and dark features [7,15]. Ideally, the smoothed image should eliminate the bright spots and fill the dark holes in the original, facilitating the extraction of both bright and dark features from the resultant difference image between the original and the smoothed version. To fulfill this objective, our local-extrema-driven image filter is constructed as follows.

Initially, we reconstruct the input image using its local minima, expressed as:

(1) $$F^{\prime }=H*I_{min},$$

where ∗ represents the convolution operator. $I_{min}$ represents the local minimum image derived from the input image *I*, calculated according to Equation (2). Additionally, *H* represents the convolution kernel, the format of which is defined in Equation (3).

(2) $$I_{min}=imerode\left(I,se\right),$$

where imerode represents the morphological erosion operator and se denotes a disk-shaped structural element with a radius *r*. Consequently, $I_{min}$ signifies the local minimum image of *I* with respect to a distance of *r*.

(3) $${\left[\begin{array}{ccccc} 1 & 1 & \cdots & 1 & 1\\ 1 & 0 & \cdots & 0 & 1\\ \vdots & \vdots & \ddots & \vdots & \vdots \\ 1 & 0 & \cdots & 0 & 1\\ 1 & 1 & \cdots & 1 & 1 \end{array}\right]}_{\left(2r+1)\times (2r+1\right)}.$$

In this manner, every pixel in the original input image is reconstructed based on the local minima of its neighboring pixels, effectively suppressing the bright features present in the original image. Subsequently, the initially filtered image $F^{\prime }$ undergoes further reconstruction, this time utilizing its local maxima, as follows:

(4) $$F=H*F_{max}^{\prime },$$

where $F_{max}^{\prime }$ represents the local maximum image derived from the initially filtered image $F^{\prime }$ and can be computed using Equation (5).

(5) $$F_{max}^{\prime }=imdilate\left(F^{\prime },se\right),$$

where imdilate signifies the morphological dilation operator. Consequently, $F_{max}^{\prime }$ represents the local maximum image of $F^{\prime }$ with a distance of *r*.

In contrast to Equation (1), Equation (4) achieves additional removal of salient dark features from the filtered image by reconstructing each pixel in $F^{\prime }$ based on its local maxima.

To streamline the presentation of the upcoming image-fusion method, we introduce $lextremefilter\left(\cdot \right)$ as the function of our devised local-extrema-driven image filter, composed of Equations (1) and (4). The process of smoothing an image with our local-extrema-driven image filter can be succinctly expressed as:

(6) $$F=lextremefilter\left(I,r\right),$$

where *r* denotes the size of the structuring element in Equations (2) and (5).

As is evident, an image comprises both bright and dark features, illustrated by the bright person and the dark window corner in Figure 1. Through the iterative reconstruction of the input image based on the local minima and local maxima, salient bright and dark features can be effectively eliminated, resulting in a well-smoothed image (see the filtered images in the last column of Figure 1). Subsequently, the salient features of the input image can be derived by subtracting the filtered image *F* from the input image *I* as per Equation (7). The positive part *B* captures the bright features (refer to the first column of the Feature Extraction and Fusion Module in Figure 1), while the negative part *D* corresponds to the dark features (refer to the second column of the Feature Extraction and Fusion Module in Figure 1).

(7) $$\left\{\begin{array}{c} B=\max\left(I-F,0\right)\\ D=\min\left(I-F,0\right) \end{array}\right.,$$

where *B* and *D* represent the bright and dark feature map of *I*, respectively.

Furthermore, the local-extrema-driven image filter can be scaled to multiple levels through successive applications of the filter driven by local minima and local maxima on the input image *I*, as outlined in Equation (8).

(8) $$F_{i}=lextremefilter\left(F_{\left(i-1\right)},r_{i}\right),$$

where *i* represents the current scale of the image filter, with *i* incrementing from 1 to *n* sequentially. $F_{i}$ denotes the filtered image at the *i*th scale, and notably, $F_{0}$ corresponds to the original input image *I*. The parameter $r_{i}$ denotes the size of the structuring element and convolution kernel at the *i*th scale. In this study, we designate $r_{i}=i$ to progressively augment the smoothing degree of our proposed image filter.

Consequently, multiple scales of bright and dark feature maps can be concurrently extracted from the continuously filtered images by

(9) $$\left\{\begin{array}{c} B_{i}=\max\left(F_{i-1}-F_{i},0\right)\\ D_{i}=\min\left(F_{i-1}-F_{i},0\right) \end{array}\right..$$

Finally, the last scale of the filtered image is taken as the base image for *I*:

(10) $$I_{base}=F_{n},$$

where *n* represents the scale number.

### 2.2. Local-Extrema-Driven Image Fusion

In this study, our objective is to fuse a visual image denoted as $I^{vis}$ and an infrared image denoted as $I^{inf}$. Utilizing the feature-extraction method outlined in the preceding subsection, multi-scale bright feature maps (represented by $B_{i}^{vis}$ and $B_{i}^{inf}$) and dark feature maps (indicated by $D_{i}^{vis}$ and $D_{i}^{inf}$) are effectively extracted from $I^{vis}$ and $I^{inf}$. Concurrently, we obtain their respective base images denoted as $I_{base}^{vis}$ and $I_{base}^{inf}$. The subsequent contents delineate the detailed procedures for fusing a visual image and an infrared image.

Considering that high-frequency bright features usually correspond to sharp and bright features in the image, we combine each scale of bright feature maps from the infrared and visual images by choosing their elementwise maximum values. Likewise, for each scale of dark feature maps, we fuse them using their elementwise minimum values. The mathematical expressions for fusing high-frequency bright and dark features are as follows:

(11) $$\left\{\begin{array}{c} B_{i}^{fuse}=\max\left(B_{i}^{vis},B_{i}^{inf}\right)\\ D_{i}^{fuse}=\min\left(D_{i}^{vis},D_{i}^{inf}\right) \end{array}\right..$$

Furthermore, the elementwise-fused bright and dark feature maps are individually integrated into single feature maps. As feature maps may contain varied quantities of features across different scales, potentially leading to redundancy, this study employs a two-step process. Initially, the strengths of these feature maps are dynamically adjusted based on their information content. Subsequently, they are summed together. This adaptation relies on an entropy-based weighting strategy [32], enhancing feature maps with a substantial amount of information while diminishing those with relatively less information. The detailed aggregation of the fused multiple scales of bright and dark feature maps is outlined below.

(12) $$\left\{\begin{array}{c} B^{fuse}=\sum_{i=1}^{n}w_{b,i}\times B_{i}^{fuse}\\ D^{fuse}=\sum_{i=1}^{n}w_{d,i}\times D_{i}^{fuse} \end{array}\right.,$$

where $w_{b,i}$ and $w_{d,i}$ denote the weights of the bright feature map and dark feature map at the *i*th scale, respectively, and can be calculated as follows:

(13) $$\left\{\begin{array}{c} w_{b,i}=\frac{e_{b,i}}{\frac{1}{n}\sum_{j=1}^{n}e_{b,j}}\\ w_{d,i}=\frac{e_{d,i}}{\frac{1}{n}\sum_{j=1}^{n}e_{d,j}} \end{array}\right.,$$

where $e_{b,i}$ and $e_{d,i}$ represent the entropy of $B_{i}^{fuse}$ and $\left(-D_{i}^{fuse}\right)$, respectively. This exploited feature aggregation strategy ensures that the fused single bright feature map and dark feature map not only retain the salient high-frequency features, but also eliminate redundant information.

Concerning the low-frequency base images, they commonly contain large-scale structural features, and the intensity distribution of the fused base image plays a crucial role in determining the final appearance of the fusion image. Therefore, in this study, we employed a structural similarity- and intensity-based scheme to fuse the base images of infrared and visual images. Specifically, we initiate the process by averaging the two base images elementwise, yielding an initial base image as follows:

(14) $${I^{\prime }}_{base}^{fuse}=0.5\times \left(I_{base}^{vis}+I_{base}^{inf}\right).$$

Subsequently, a provisional fusion image ${I^{\prime }}^{fuse}$ is created by combining the fused bright feature map, fused dark feature map, and initially fused base image as follows:

(15) $${I^{\prime }}^{fuse}=B^{fuse}+D^{fuse}+{I^{\prime }}_{base}^{fuse}.$$

Afterward, the structural-similarity maps between each base image and the initially fused image are computed, respectively.

(16) $$\left\{\begin{array}{c} S^{vis}=SSIM\left({I^{\prime }}^{fuse},I_{base}^{vis}\right)\\ S^{inf}=SSIM\left({I^{\prime }}^{fuse},I_{base}^{inf}\right) \end{array}\right.,$$

where $SSIM\left(A,B\right)$ calculates the structural similarity between image *A* and image *B* using the method outlined in [33]. Afterward, we generate a structural similarity-based weight map for fusing base images as follows:

(17) $$w_{base,vis}^{struct}=S^{vis}/\left(S^{vis}+S^{inf}\right).$$

Moreover, the grayscale intensities are closely linked to the appearance of the fusion image. Therefore, we also incorporate an intensity-based weight for fusing base images, which can be computed as follows:

(18) $$w_{base,vis}^{intens}=e^{I^{vis}/\left(I^{vis}+I^{inf}\right)}.$$

To balance the two kinds of weights, we fuse them by

(19) $$w_{base,vis}=G*\left[w_{base,vis}^{struct}\times {\left(w_{base,vis}^{intens}\right)}^{\alpha }\right],$$

where α serves as a parameter to balance these two weights. *G* represents a Gaussian kernel employed to smooth the weight distribution map.

Then, the two base images of the infrared and visual images can be fused as follows:

(20) $$I_{base}^{fuse}=w_{base,vis}\times I_{base}^{vis}+\left(1-w_{base,vis}\right)\times I_{base}^{inf}.$$

**Figure 2:**
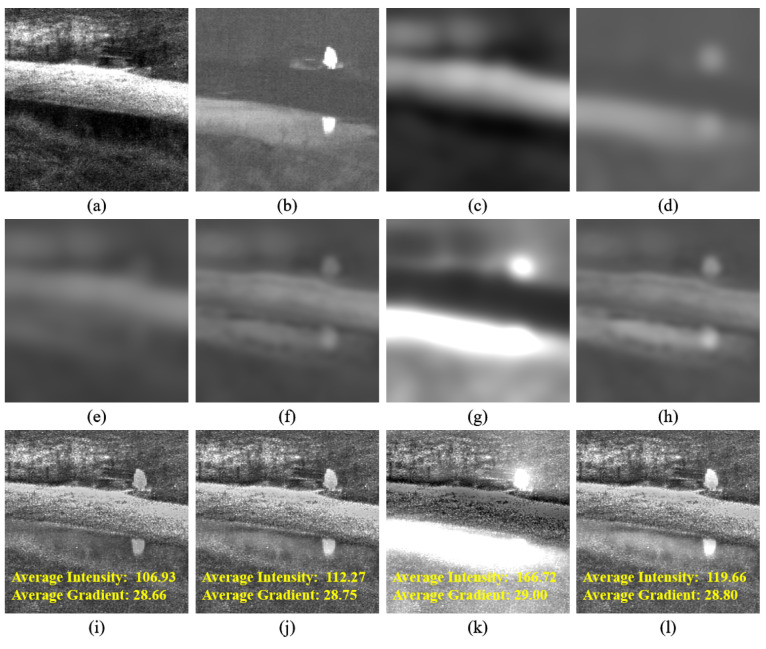
Demonstration example of our base image fusion scheme. (**a**,**b**) present the original visual and infrared images, respectively. (**c**,**d**) depict the base images corresponding to the infrared and visual inputs, respectively. (**e**–**h**) exhibit the resulting fused base images derived from the direct average scheme, structural similarity-based fusion, intensity-based fusion, and our novel structural similarity- and intensity-based fusion approach, respectively. (**i**–**l**) showcase the fusion images generated by combining (**e**–**h**) with our fused high-frequency bright and dark feature maps, respectively. The yellow text in (**i**–**l**) highlights the average grayscale intensity and average absolute gradient of the corresponding fused image.

As depicted in Figure 2, the implementation of our structural similarity- and intensity-based fusion scheme results in a fused base image that not only retains significant large-scale structural features from both base images, but also achieves an advantageous intensity distribution, thereby enhancing visual perception in the final fusion image. Specifically, when compared to exclusively utilizing the structural similarity-based fusion scheme (see Figure 2f), our comprehensive fusion scheme produces a fused base image (see Figure 2h) with a more suitable intensity distribution. Similarly, in contrast to relying solely on an intensity-based fusion scheme (see Figure 2g), our comprehensive fusion approach retains a greater number of structural features in the fused base image (see Figure 2h). Furthermore, compared to simply averaging the two base images (see Figure 2e), our complete base image-fusion scheme generates an intensity-distributed fused base image (see Figure 2h) while preserving richer textures. Additionally, by comparing the fusion images generated from the fused base images in Figure 2e,f, it effectively validates the efficacy of our base image fusion scheme to a significant extent.

Finally, our proposed method generates the fusion image by combining the fused bright feature map, dark feature map, and base image together, as expressed in Equation (21). Through this process, our fused image not only retains fundamental information from the infrared and visual images, but also effectively highlights the prominent sharp features present in the infrared and visual images.

(21) $$I^{fuse}=B^{fuse}+D^{fuse}+I_{base}^{fuse}.$$

### 2.3. Parameter Settings

The proposed method involves two parameters: the scale number *n* and the parameter α for balancing $w_{base,vis}^{struct}$ and $w_{base,vis}^{intens}$. In this study, we employed the grid search method to find the optimal pair of *n* (ranging from 1 to 10 in increments of 1) and α (ranging from 0.05 to 1 in increments of 0.05) that maximizes the multi-scale structural similarity metric (MSSIM) [34]. The results show that the MSSIM increases with the increase of the scale number, but the running time of our method increases simultaneously. So, we first set the scale number *n* to six, so that the performance and time cost of our method will be balanced. Afterwards, when n=6, MSSIM is maximized by setting α=0.35. Therefore, throughout this study, consistent parameter settings (n=6 and α = 0.35) were used, and the experimental results in the following section validate the efficacy of these chosen parameters for infrared and visual image fusion.

## 3. Experimental Results and Discussion

To showcase the merits of our novel infrared and visual image-fusion method, we conducted a thorough comparative analysis against eleven state-of-the-art image-fusion techniques. This evaluation was performed on a widely recognized dataset for infrared and visual images. For comprehensive insights into the experimental settings, results, and discussions, please refer to the subsequent subsections.

### 3.1. Experimental Settings

The experimental setup for this study is summarized as follows. Initially, we assembled twenty pairs of widely used infrared and visual images from the TNO dataset [35]. Subsequently, we selected eleven state-of-the-art image-fusion methods for comparison. These methods include the guided-filter-based image method (GFF) [36], the hybrid multi-scale-decomposition-based image-fusion method (HMSD) [18], the Laplacian pyramid- and sparse-representation-based image-fusion method (LPSR) [25], the Gaussian of differences-based image-fusion method (GDPSQCV) [37], the relative total variation-decomposition-based image-fusion method (RTVD) [38], the parameter-adaptive unit-linking dual-channel PCNN-based image-fusion method (PAULDCPCNN) [39], the GAN-based image-fusion method (FusionGAN) [16], the unified deep learning-based image-fusion method (U2Fusion) [40], the semantic-aware image-fusion method (SeAFusion) [28], and the representation learning-guided image-fusion method (LRR) [29]. For simplicity, we refer to our proposed local-extrema-driven filter-based image-fusion method as LEDIF. Additionally, we conducted comparisons by excluding the utilization of the structural similarity- and intensity-based base image fusion scheme in our method (denoted as ${\mathrm{LEDIF}}_{0}$) to evaluate the effectiveness of this scheme.

Afterwards, the thirteen methods underwent both qualitative and quantitative evaluation. In particular, the qualitative assessment involved a visual comparison of the fusion results across the different methods. For the quantitative evaluation, we employed nine metrics to objectively gauge the quality of the fusion images produced by the various approaches. These metrics include the spatial frequency (SF) [8,41], the average absolute gradient (AG) [42], the linear index of fuzziness (LIF) [43], the blind/referenceless image spatial quality evaluator (BRISQUE) [44], the visual information fidelity (VIF) [45], the multi-scale structural similarity index metric (MSSIM) [34], the edge-dependent structural similarity index metric (ESSIM) [46], the edge-similarity-based metric (QABF) [44] and the sum of correlation differences metric (SCD) [47]. The superior performance of the corresponding image-fusion method is indicated by smaller values for the BRISQUE metric and larger values for the other eight metrics.

Among these metrics, the SF, AG, and LIF quantify the amount of details preserved in the fusion image, while BRISQUE quantifies the clarity and distortion level of the fusion image. The VIF measures the information fidelity of the fusion image concerning the input images, while the MSSIM, ESSIM, QABF, and SCD gauge the structural similarity between the fusion image and the input images from various perspectives. These metrics collectively provide a comprehensive evaluation framework, capturing different aspects of fusion image quality and fidelity.

### 3.2. Qualitative Evaluation Results

**Figure 3:**
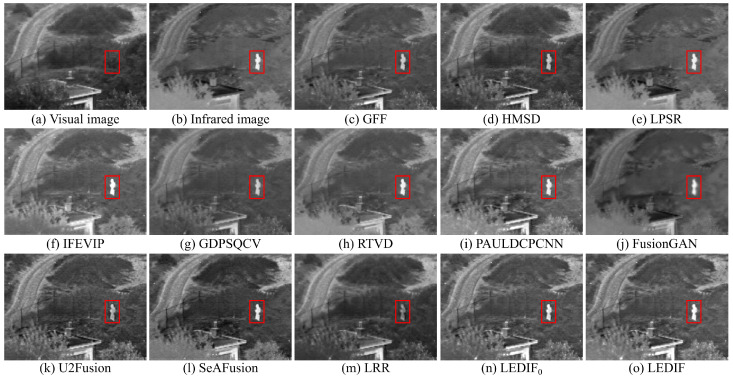
First comparison example of the thirteen image-fusion methods.

**Figure 4:**
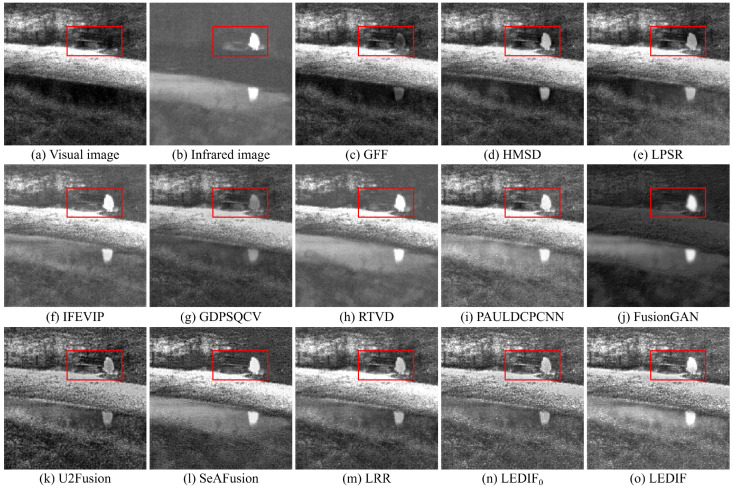
Second comparison example of the thirteen image-fusion methods.

**Figure 5:**
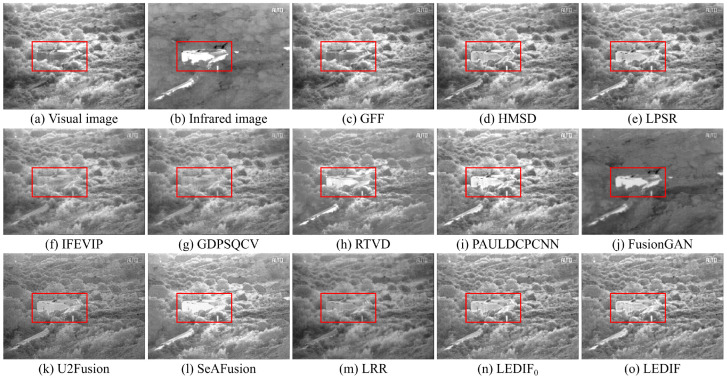
Third comparison example of the thirteen image-fusion methods.

**Figure 6:**
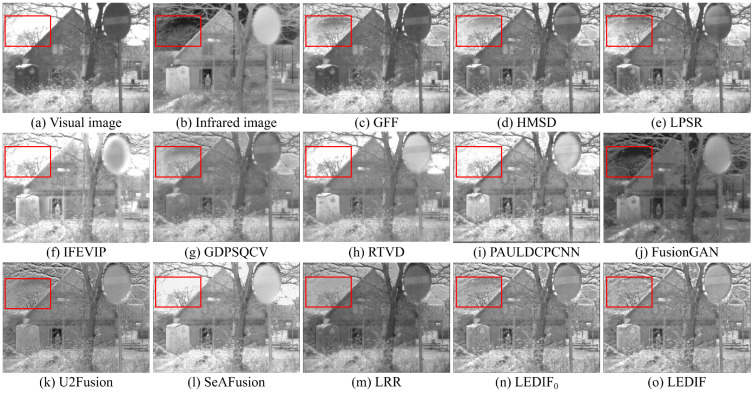
Fourth comparison example of the thirteen image-fusion methods.

**Figure 7:**
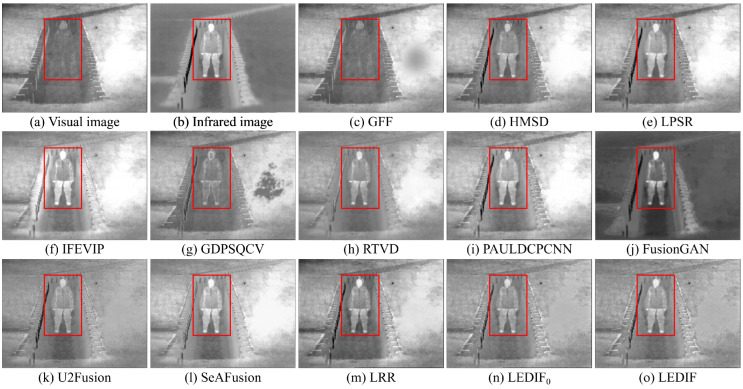
Fifth comparison example of the thirteen image-fusion methods.

In this subsection, we qualitatively assess the thirteen image-fusion methods by visually comparing their fusion results. To offer visual insight into the quality and effectiveness of each fusion method, we present five comparison examples showcasing the fusion outputs of all thirteen methods in Figure 3, Figure 4, Figure 5, Figure 6 and Figure 7, respectively.

In Figure 3, both the infrared and visual images were captured under normal lighting conditions. Notably, a person was standing near the fence, appearing almost invisible in the visual image while prominently visible in the infrared counterpart. Consequently, an ideal fusion image for this image pair should seamlessly integrate the bright person and distinct spots from the infrared image with the intricate textures of the trees and fence from the visual image. It is evident that the areas corresponding to the person in the fusion images produced by the GFF, HMSD, GDPSQCV, U2Fusion, and LRR in (c), (d), (g), (k), and (m) appear dimmer compared to those in other fusion images. Similarly, the tree regions in the fusion images generated by the LPSR, IFEVIP, GDPSQCV, RTVD, and FusionGAN in (e), (f), (g), (h), and (j) exhibit relatively smoother textures than those in other fusion images. Notably, the intensities in the fusion image of PAULDCPCNN, as depicted in (i), are not evenly distributed. Additionally, the background of the fusion image produced by SeAFusion, illustrated in (l), appears noticeably darker compared to others. Finally, (n) and (o) demonstrate that our two fusion images exhibit the most visually appealing results among all fusion images, with the fusion image generated by our complete method in (o) being slightly brighter than that produced by our method without leveraging the proposed base image fusion scheme.

In Figure 4, the infrared and visual images were captured under low-light conditions. The optimal fusion image for this pair should seamlessly integrate distinctive bright features, particularly the two person regions in the infrared image, and the bright textures of the visual image, encompassing the grass and trees, along with the darker features represented by the bench. Among the fusion images depicted in (c), (e), (g), (k), (m), and (n), generated by the GFF, LPSR, GDPSQCV, U2Fusion, LRR, and our ${\mathrm{LEDIF}}_{0}$, respectively, the intensities of the person regions are notably lower than those in (b), indicating unsatisfactory results in this particular case. Furthermore, the contrast in the fusion results of GDPSQCV and U2Fusion in (g) and (k) is relatively diminished compared to other methods’ fusion images. The fusion image of RTVD in (h) is over-exposed, resulting in the loss of many textural details, particularly around the bench. Conversely, the fusion image of FusionGAN in (j) fails to integrate most critical textures of the visual image in (a). While the HMSD, IFEVIP, SeAFusion, and our LEDIF in (d), (f), (l), and (o), respectively, exhibit the most visually appealing results among all fusion images, there are notable observations. IFEVIP’s fusion image in (f) appears slightly over-exposed, and the bright infrared features of the HMSD’s fusion image in (d) are relatively lower than other methods’ results. Additionally, both the IFEVIP and SeAFusion sacrifice some textural details in their fusion images in (f) and (l). In summary, the fusion image generated by our LEDIF in (o) attains the highest visual quality, affirming the effectiveness of our structural similarity- and intensity-based base image fusion scheme in enhancing the overall visual appearance of the final fusion images.

In Figure 5, both the infrared and visual images were captured under normal lighting conditions. The ideal fusion image should effectively combine the various scales of salient bright features from the infrared image with the diverse bright and dark features present in the visual image. It is evident from (c), (f), (g), and (m) that the GFF, IFEVIP, GDPSQCV, and LRR struggle to integrate most of the bright features from the infrared image into their fusion images, as observed in the building area within the red bounding boxes of each image. Among these methods, FusionGAN’s fusion image in (f) displays a considerable loss of textures from the visual image, resulting in the poorest visual effect among all thirteen image-fusion methods. U2Fusion manages to integrate the salient features of both the infrared and visual images into its fusion image, as demonstrated in (k). However, the contrast of (k) is relatively low compared to that of the infrared image, the visual image, and most other fusion images. (l) highlights that the building area of the fusion image generated by SeAFusion is over-exposed, leading to a loss of some building details. Ultimately, the fusion images produced by PAULDCPCNN, our ${\mathrm{LEDIF}}_{0}$, and our LEDIF in (i), (n), and (o), respectively, exhibit the most favorable visual effects among all fusion images.

In Figure 6, the sky area in the visual image appears over-exposed, necessitating an ideal fusion image for this image pair to accentuate the bright tree features surrounding the sky area from the infrared image. In (c), the GFF demonstrates limitations in incorporating the bright person from the infrared image into its fusion image. While the HMSD and LPSR effectively blend the infrared and visual images in most regions, they struggle to integrate specific bright tree branches from the infrared image, as highlighted in the red bounding boxes of (d) and (e). Moving on to (f), (h), (i), and (l), the IFEVIP, RTVD, PAULDCPCNN, and SeAFusion encounter challenges in including the bright tree branches from the infrared image in their fusion images due to the over-exposed sky area in the visual image. Conversely, the fusion images from the GDPSQCV, FusionGAN, U2Fusion, and LRR in (g), (j), (k), and (m) exhibit the loss of textural details from the visual image, with relatively low contrast compared to other methods. Furthermore, (n) and (o) illustrate that the fusion images generated by the PAULDCPCNN, our ${\mathrm{LEDIF}}_{0}$, and LEDIF in (i), (n) and (o) successfully integrate the bright tree branches from the infrared image, displaying good contrast compared to the fusion images from the other methods. Notably, the fusion image from our LEDIF is slightly brighter than that of our ${\mathrm{LEDIF}}_{0}$, indicating a slight improvement in the visual effect of the fusion image facilitated by the proposed base image fusion scheme in this case.

In Figure 7, both the infrared and visual images were captured under low-light conditions. The primary goal for this pair was to generate an optimal fusion image that effectively integrates the facial features depicted in the visual image in (a) with the bright person captured in the infrared image in (b). (c) reveals that the GFF fails to effectively integrate the bright person features from the infrared image into its fusion image. Notably, the fusion images of the HMSD, U2Fusion, and our ${\mathrm{LEDIF}}_{0}$ in (d), (k), and (n), respectively, exhibit relatively low contrast compared to other fusion images. Furthermore, (f), (h), and (i) demonstrate that the fusion images of the IFEVIP, RTVD, and SeAFusion appear over-exposed, resulting in a failure to integrate most facial features from the visual image. In (g), the fusion image generated by the GDPSQCV showcases a significant loss of the person area, while (j) indicates that most background areas of FusionGAN’s fusion image fail to integrate from the visual image. Overall, in this scenario, fusion the images obtained from the LPSR, PAULDCPCNN, LRR, and our LEDIF in (e), (i), (m), and (o), respectively, achieve the most favorable visual effects.

The qualitative comparisons across the five examples strongly affirm the efficacy of our proposed method in seamlessly integrating the prominent bright and dark features present in both infrared and visual images, resulting in comprehensive fusion images. Notably, our method consistently performed comparably or even surpassed eleven state-of-the-art image fusion approaches, as evidenced by superior visual observations. Additionally, the visual comparison examples further validate the effectiveness of our proposed base image fusion scheme in enhancing the visual quality of the fusion images.

### 3.3. Quantitative Evaluation Results

As widely acknowledged, qualitative evaluation heavily depends on subjective observation, potentially resulting in inaccuracies and demanding significant effort. To ensure an objective comparison of the performance of various methods, we additionally utilized nine quantitative metrics, as outlined at the beginning of this section. Subsequently, we provide detailed quantitative evaluation results and discussions.

**Figure 8:**
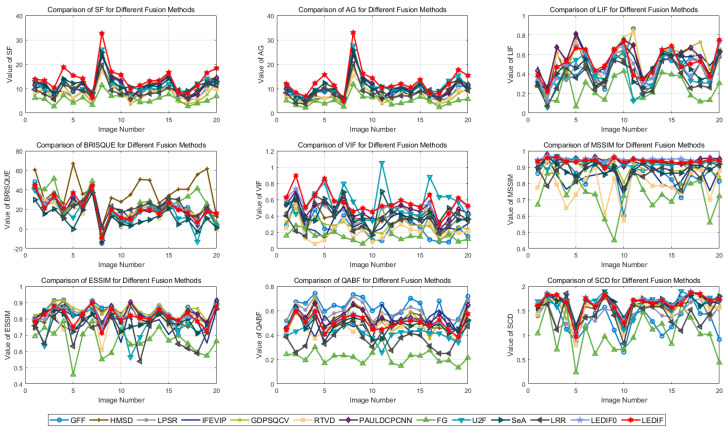
Visual comparison of the quantitative evaluation results.

Table 1 presents the quantitative metrics computed for the thirteen image-fusion methods. Notably, in Table 1, the best, second-best, and third-best values are highlighted in red, green, and blue, respectively, while the integer in the subscript of each metric value indicates the performance rank among all thirteen image-fusion methods. Additionally, the individual metric values for each fusion image generated by each method are further illustrated in Figure 8.

The analysis of the metrics reveals that our proposed method achieved top performance on two metrics, the SF and AG, while securing the second-best performance on the VIF metric and the third-best performance on the MSSIM and SCD metrics. Furthermore, our method ranked in the top 50% for the other four metrics, including the LIF, BRISQUE, ESSIM, and QABF. Specifically, our method stands out with the largest SF and AG values and the fifth-largest LIF value, indicating superior preservation of textural details compared to the other twelve comparison methods. Regarding BRISQUE, our method ranked seventh, suggesting relatively high-quality image generation with clarity and information retention.

Additionally, our method ranked second on the VIF, indicating high visual information fidelity with respect to the original visual images. In terms of the MSSIM, our ${\mathrm{LEDIF}}_{0}$ and LEDIF ranked first and third, respectively, on this metric. The MSSIM, being a multi-scale structural similarity measure, is often more robust than other similarity measures like the ESSIM and QABF, where our method ranked seventh. These structural similarity-based metrics validate our method’s ability to preserve relatively more structural features from the input infrared and visual images. Similarly, our method ranked third on the SCD metric, indicating close correlation between the fusion images and the original infrared and visual images, thereby preserving more structural features.

Furthermore, comparing the metric values of our ${\mathrm{LEDIF}}_{0}$ and LEDIF reveals that the LEDIF preserved more details from the input images in its fusion images compared to ${\mathrm{LEDIF}}_{0}$, as inferred from the SF, AG, and LIF metrics. The LEDIF also generated fusion images of higher visual quality and fidelity, as indicated by the BRISQUE and VIF metrics. However, incorporating the base image fusion scheme resulted in a slight loss of structural features compared to ${\mathrm{LEDIF}}_{0}$, evident from metrics like the MSSIM, ESSIM, QABF, and SCD.

The consistency between the average metrics and individual values is further validated by the individual metric values plotted in Figure 8. This consistency reinforces the effectiveness and significance of the quantitative ranks discussed above.

### 3.4. Further Discussion

When compared to existing or related methods, in particular the approach presented in [31], our method stands out significantly. While both methods rely on a local image filter, the method in [31] is constructed based on the original Bezier interpolation operation, which differs from our construction method. Additionally, the cited method does not address the enhancement of visual quality in the final fusion images. In contrast, our method specifically tackles this issue, particularly addressing the challenge of dim visual effects in fusion images by introducing a novel intensity and structural similarity-based base image fusion scheme. Through both qualitative and quantitative analyses, our newly proposed local-extrema-filter-based image-fusion method and base image fusion scheme prove to be effective for infrared and visual image fusion tasks, performing comparably to or even better than eleven state-of-the-art image-fusion methods.

Furthermore, the efficiency of our image-fusion method is relatively high, requiring approximately 0.21 s to fuse a pair of infrared and visual images. Nevertheless, there exists substantial potential for further efficiency enhancements through the utilization of parallel computing techniques or the optimization of computational operations. Therefore, there is great potential to apply our proposed method to real practical scenarios.

**Figure 9:**
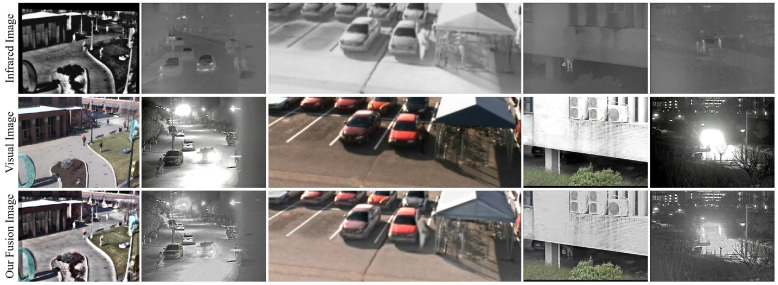
More results of our method for fusing images from other infrared and visual image fusion dataset (i.e., the VIFB dataset [48]).

To comprehensively evaluate the generalization ability of our method, we first conducted experiments using the VIFB dataset [48]. The results, depicted in Figure 9, showcase five representative image fusion examples. These examples not only demonstrate our method’s capability to fuse images captured under varying lighting conditions, including both daylight and nighttime scenarios, but also its effectiveness in seamlessly integrating salient infrared features with over-exposed visual images.

**Figure 10:**
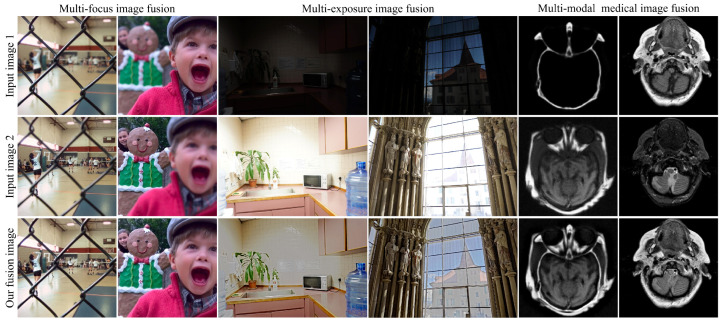
More results of our method for fusing multi-focus, multi-exposure, and multi-modal medical images.

Expanding beyond infrared–visual fusion, our method was applied to fuse images from diverse modalities, including multi-focus images, multi-exposure images, and multi-modal medical images. As depicted in Figure 10, our approach adeptly integrates salient features from each pair of source images into the resulting fusion images. This versatility underscores the adaptability and robustness of our method across a wide range of image modalities.

In summary, the positive fusion results observed in both Figure 9 and Figure 10 serve as compelling validation of the robust generalization ability of our method. Its efficient processing time, combined with its effectiveness across varied modalities, positions our approach as a promising solution for real-world image-fusion applications. Through ongoing research and refinement, we remain committed to further advancing the capabilities and applicability of our method in diverse image-fusion scenarios.

Considering both qualitative and quantitative evaluations, our image-fusion method consistently demonstrates performance on par with or superior to the eleven state-of-the-art image-fusion methods.

## 4. Conclusions

In this study, we have introduced a highly effective local-extrema-driven image filter, meticulously designed for the fusion of infrared and visual images. The proposed filter showcases remarkable capabilities in smoothing images, thereby facilitating the extraction of salient bright and dark features. Through iterative application of this filter, our approach excels at extracting multiple scales of salient textural features from both infrared and visual images. These distinctive features are seamlessly integrated into a single, informative fusion image through two appropriate fusion strategies. Notably, our innovative base image fusion scheme, rooted in structure similarity and intensity, significantly enhances the visual effect of the resulting fusion images.

While our method demonstrates competitive performance against state-of-the-art techniques, several avenues for further research and improvement are apparent. Primarily, the current reliance on grid searching for parameter optimization may not yield the most optimal settings for the infrared and visual image fusion task. To address this limitation, we intend to explore advanced optimization techniques to fine-tune these parameters, ensuring maximal performance and adaptability across diverse datasets and scenarios.

Furthermore, although our method excels in enhancing low-level image features, its current configuration lacks optimization for high-level vision tasks such as image segmentation, object detection, and object tracking. Recognizing the significance of seamlessly integrating these capabilities, our future research endeavors will focus on evolving our framework into a deep learning-driven architecture. By harnessing the power of deep learning, we aim to imbue our method with the capacity to not only preserve critical image features during fusion, but also to facilitate robust performance in subsequent high-level vision tasks, thereby enhancing its utility and applicability in real-world surveillance systems.

Moreover, while our base image fusion scheme yields visually appealing results, we acknowledge its marginal impact on certain quantitative metrics. To address this, we plan to explore novel fusion strategies and evaluation metrics that better capture the holistic quality and utility of fusion images. By refining our approach in this manner, we aim to bridge the gap between subjective visual appeal and objective performance metrics, thereby ensuring a comprehensive assessment of fusion image quality.

In summary, while our method presents a significant advancement in the field of image fusion, we recognize the importance of continuous refinement and adaptation to meet the evolving demands of contemporary surveillance systems. Through targeted research efforts aimed at parameter optimization, the integration of high-level vision tasks, and the refinement of fusion strategies, we are committed to further enhancing the capabilities and applicability of our approach for diverse real-world scenarios.

## Figures and Tables

**Table 1:** Quantitative evaluation results of different image-fusion methods on the datasets used.

Methods	SF	AG	LIF	BRISQUE	VIF	MSSIM	ESSIM	QABF	SCD
GFF	$10.6666_{8}$	$9.1331_{8}$	$0.4448_{9}$	$20.7646_{8}$	$0.2521_{11}$	$0.8558_{10}$	$0.8418_{3}$	$0.6218_{1}$	$1.2982_{12}$
HMSD	$11.7816_{4}$	$10.2579_{5}$	$0.4493_{8}$	$38.3982_{13}$	$0.3976_{7}$	$0.9324_{4}$	$0.8319_{5}$	$0.5330_{4}$	$1.5675_{8}$
LPSR	$11.2857_{7}$	$9.8681_{7}$	$0.4333_{10}$	$19.0110_{4}$	$0.4065_{6}$	$0.9289_{5}$	$0.8428_{2}$	$0.5931_{2}$	$1.4170_{12}$
IFEVIP	$9.5708_{9}$	$8.3164_{10}$	$0.5502_{2}$	$21.9260_{10}$	$0.3231_{9}$	$0.8482_{11}$	$0.7740_{11}$	$0.4981_{8}$	$1.6437_{4}$
GDPSQCV	$8.2206_{12}$	$6.9785_{12}$	$0.5343_{4}$	$20.1511_{6}$	$0.2766_{10}$	$0.8929_{7}$	$0.8557_{1}$	$0.5078_{5}$	$1.5771_{7}$
RTVD	$8.4358_{11}$	$7.2621_{11}$	$0.5433_{3}$	$19.2725_{5}$	$0.2122_{12}$	$0.7893_{12}$	$0.7878_{8}$	$0.4609_{10}$	$1.5386_{9}$
PAULDCPCNN	$11.3139_{6}$	$9.9305_{6}$	$0.5565_{1}$	$18.9329_{3}$	$0.4707_{4}$	$0.9412_{2}$	$0.8330_{4}$	$0.5409_{3}$	$1.6402_{5}$
FusionGAN	$5.7691_{13}$	$5.0467_{13}$	$0.2478_{13}$	$25.4100_{12}$	$0.1831_{13}$	$0.7308_{13}$	$0.6647_{13}$	$0.2196_{13}$	$1.0213_{13}$
U2Fusion	$11.3629_{5}$	$10.6915_{4}$	$0.4095_{11}$	$16.9311_{2}$	$0.5758_{1}$	$0.9250_{6}$	$0.7809_{10}$	$0.4241_{11}$	$1.6326_{6}$
SeAFusion	$11.9697_{3}$	$10.7101_{3}$	$0.4536_{7}$	$10.8181_{1}$	$0.4367_{5}$	$0.8863_{8}$	$0.7862_{9}$	$0.4761_{9}$	$1.6687_{2}$
LRR	$9.4230_{10}$	$8.4783_{9}$	$0.3652_{12}$	$23.2087_{11}$	$0.3552_{8}$	$0.8709_{9}$	$0.7429_{12}$	$0.3735_{12}$	$1.4358_{10}$
${\mathrm{LEDIF}}_{0}$	$14.1944_{2}$	$12.6043_{2}$	$0.4898_{6}$	$21.2551_{9}$	$0.5468_{3}$	$0.9478_{1}$	$0.8176_{6}$	$0.5015_{6}$	$1.6739_{1}$
LEDIF	$14.2382_{1}$	$12.6777_{1}$	$0.5165_{5}$	$20.7572_{7}$	$0.5661_{2}$	$0.9375_{3}$	$0.8141_{7}$	$0.4986_{7}$	$1.6484_{3}$

## Data Availability

The code of our image-fusion method and dataset used in this study will be released at https://github.com/uzeful/LEDIF.
